# SNP rs10420324 in the AMPA receptor auxiliary subunit TARP γ-8 regulates the susceptibility to antisocial personality disorder

**DOI:** 10.1038/s41598-021-91415-9

**Published:** 2021-06-07

**Authors:** Shi-Xiao Peng, Yue-Ying Wang, Min Zhang, Yan-Yu Zang, Dan Wu, Jingwen Pei, Yansong Li, Jiapei Dai, Xiaoyun Guo, Xingguang Luo, Ning Zhang, Jian-Jun Yang, Chen Zhang, Xiang Gao, Na Liu, Yun Stone Shi

**Affiliations:** 1 MOE Key Laboratory of Model Animal for Disease Study, Department of Neurology, Drum Tower Hospital, Medical School, Nanjing University, Nanjing, 210032 China; 2 State Key Laboratory of Pharmaceutical Biotechnology, Model Animal Research Center, National Resource Center for Mutant Mice, Medical School, Nanjing University, Nanjing, 210032 China; 3 School of Psychology, Nanjing Normal University, Nanjing, 210029 China; 4 Reward, Competition and Social Neuroscience Lab, Department of Psychology, School of Social and Behavioral Sciences, Nanjing University, Nanjing, 210023 China; 5 Chinese Brain Bank Center, Wuhan, 430074 China; 6 Shanghai Mental Health Center, Shanghai Jiao Tong University School of Medicine, Shanghai, 200030 China; 7 Division of Human Genetics, Department of Psychiatry, Yale University School of Medicine, New Haven, CT 06510 USA; 8 Department of Medical Psychology, Nanjing Medical University Affiliated Nanjing Brain Hospital, Nanjing, 210029 China; 9 Department of Anesthesiology and Perioperative Medicine, First Affiliated Hospital of Zhengzhou University, Zhengzhou, 450052 Henan Province China; 10 School of Basic Medical Sciences, Beijing Key Laboratory of Neural Regeneration and Repair, Advanced Innovation Center for Human Brain Protection, Capital Medical University, Beijing, 100069 China; 11 Institute for Brain Sciences, Nanjing University, Nanjing, 210032 China; 12 Chemistry and Biomedicine Innovation Center, Nanjing University, Nanjing, 210032 China

**Keywords:** DNA, Human behaviour, Animal behaviour, Ion channels in the nervous system, Social behaviour, Gene expression

## Abstract

In the brain, AMPA receptors mediate fast excitatory neurotransmission, the dysfunction of which leads to neuropsychiatric disorders. Synaptic function of AMPA receptors is tightly controlled by a protein group called transmembrane AMPAR regulatory proteins (TARPs). TARP γ-8 (also known as CACNG8) preferentially expresses in the hippocampus, cortex and subcortical regions that are critical for emotion generation indicating its association with psychiatric disorders. Here, we identified rs10420324 (T/G), a SNP located in the human *CACNG8* gene, regulated reporter gene expression in vitro and TARP γ-8 expression in the human brain. A guanine at the locus (rs10420324G) suppressed transcription likely through modulation of a local G-quadruplex DNA structure. Consistent with these observations, the frequency of rs10420324G was higher in patients with anti-social personality disorder (ASPD) than in controls, indicating that rs10420324G in *CACNG8* is more voluntary for ASPD. We then characterized the behavior of TARP γ-8 knockout and heterozygous mice and found that consistent with ASPD patients who often exhibit impulsivity, aggression, risk taking, irresponsibility and callousness, a decreased γ-8 expression in mice displayed similar behaviors. Furthermore, we found that a decrease in TARP γ-8 expression impaired synaptic AMPAR functions in layer 2–3 pyramidal neurons of the prefrontal cortex, a brain region that inhibition leads to aggression, thus explaining, at least partially, the neuronal basis for the behavioral abnormality. Taken together, our study indicates that TARP γ-8 expression level is associated with ASPD, and that the TARP γ-8 knockout mouse is a valuable animal model for studying this psychiatric disease.

## Introduction

Human emotion arises from interactions between brain regions within the limbic system, including the amygdala, hippocampus, insula, and cingulate cortex^1–3^. Neurons from different brain areas form neural circuits through connection at highly organized intercellular structures, the synapses. The strength of synaptic transmission is dynamically regulated by neuronal activity, a process called synaptic plasticity. In the last two decades, it has been well established that the synaptic plasticity in specific neuronal circuits are related to emotional regulation^4,5^. Glutamate is the major excitatory neurotransmitter in the brain, and AMPA-type glutamate receptors (AMPARs) on the postsynaptic membrane rapidly transmit excitatory signals by opening to allow cations to enter the neuron. The trafficking of AMPARs to and from the synaptic membrane also mediates long-term plasticity of excitatory synapses^6,7^. Dysregulation of AMPAR trafficking results in impaired synaptic plasticity, abnormal neuronal networks, altered emotions or behaviors, and neuronal diseases^8–11^.

In the brain, AMPARs are homomeric or heteromeric tetramers composed of the pore-forming subunits of GluA1–4. Recently, an expanding list of transmembrane auxiliary subunits are identified to associate with the AMPAR complex, including transmembrane AMPAR regulatory protein (TARP), cornichon-like (CNIH), germ cell-specific gene 1-like(Gsg1l) and the cysteine-knot AMPAR-modulating protein (CKAMP)/Shisa protein family^12–15^, which modulate AMPAR biophysics, intracellular trafficking and synaptic expression^16–18^.

TARPs are the earliest-identified and best-characterized AMPAR auxiliary subunits. They are officially named as the calcium channel gamma subunits (CACNGs) for their homology to CACNG1. Six TARPs have been classified as type 1 (γ-2, γ-3, γ-4, and γ-8) or type 2 (γ-5 and γ-7) based on their sequence similarities and functional properties^17^. In the brain, these six proteins have different expression patterns^19^. Key insights regarding their physiological roles have been derived from studies on mutant mice. For example, the absence of the prototypical auxiliary subunit γ-2 (stargazin) diminishes excitatory synaptic transmission from cerebellar mossy fibers to granule cells^20–22^ and causes behavioral abnormalities including ataxia, head elevation^23^. Selective deletion of γ-2 and γ-7 in the cerebellar Purkinje neurons of mice results in severe disruption of motor behaviors^24^.

TARP γ-8 (CACNG8) was first reported in 2001 for its homology to TARP γ-2^25^. In γ-8 knockout mice, hippocampal AMPA receptors do not progress through the secretory pathway and are not efficiently trafficked to dendrites^26,27^, and the mice show hyperactivity and marked reductions in digging and burying behaviors^28^. High TARP γ-8 expression was detected in the cortex, hippocampus and subcortical regions^19^, brain areas that are critical for emotion generation. This expression pattern suggests that TARP γ-8 may affect emotion-related behaviors. In deeds, a large-scale association study suggests that a two-SNP haplotype (rs10420331-rs11084307) in the intronic region of *CACNG8* is associated with schizophrenia in a Chinese Han population^29^. However, whether and how these two SNPs cause emotional disorders are unclear. The exact role and the molecular mechanisms of TARP γ-8 in emotion remain elusive.

In this study, to understand whether and how TARP γ-8 regulates human emotion, we first analyzed SNPs in human *CACNG8* gene and identified SNP rs10420324 located in the first intron which controlled the expression of a reporter gene in vitro and the expression of TARP γ-8 in human brains. Then we found that rs10420324G was associated with antisocial personality disorder (ASPD) in a Chinese Han sample set. Systematic neurobehavioral tests of TARP γ-8^−/−^ and γ-8^+/−^ mice indicated that they have impaired social behaviors, including impulsion, aggression, risk ignoring, irresponsibility-like, and callousness-like, which closely mimic antisocial behaviors in humans. Therefore, our study suggests that SNP rs10420324 in *CACNG8* regulates the susceptibility to ASPD. We propose that the TARP γ-8 knockout mouse is a valuable animal model for studying ASPD.

## Results

### SNP rs10420324 in the human *CACNG8* gene regulates gene expression

**Figure 1:**
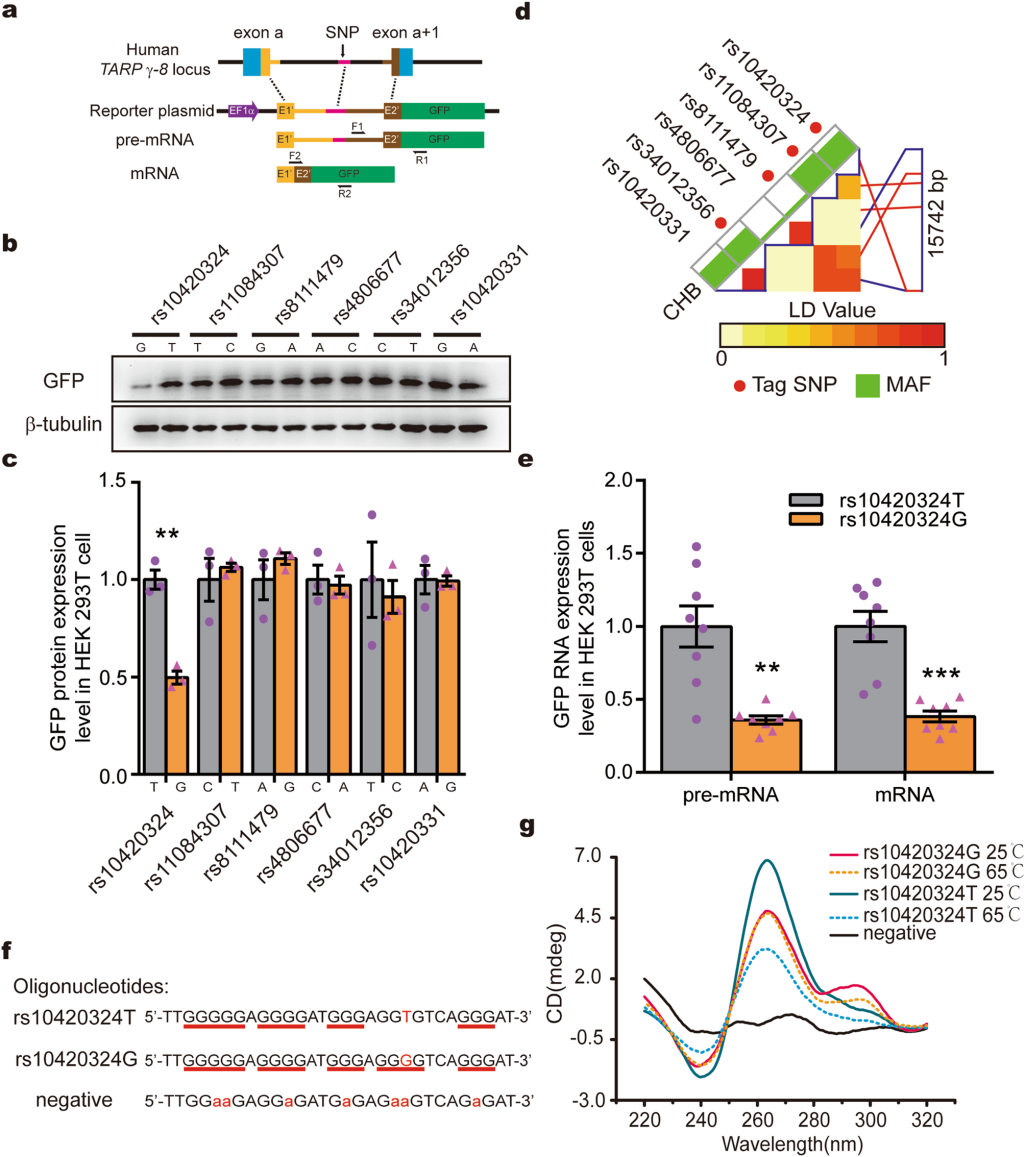
The rs10420324 SNPs regulate reporter gene expression by regulating the conformation and stability of a local G-quadruplex. (**a**) Schematic of the SNP fragment constructs inserted in the GFP reporter vector used in (**b**, **c**, **e**). The primers F1 and R1 were designed to examine pre-mRNA, while primers F2 and R2 were for mRNA. (**b**) Western blot analysis of GFP and β-tubulin protein in HEK 293 T cells transfected with the reporter plasmid. (**c**) Quantification of GFP expression levels. GFP expression was normalized using β-tubulin. (**d**) A linkage disequilibrium (LD) map for the TARP γ-8 gene was obtained from the SNPinfo web server, demonstrating a high LD value between the indicated SNPs. MAF: minor allele frequency. Red lines: Tag SNP location. Blue lines: SNP linked with Tag SNP location. (**e**) Fold change in GFP pre-mRNA and mRNA in HEK 293 T cells transfected with the reporter plasmid. GFP pre-mRNA and mRNA expression was normalized with β-actin. (**f**) Oligonucleotides containing the SNP rs10420324 were synthesized for CD spectral analysis. Underlined sequences indicate the guanine tandem repeats. An oligonucleotide with a G-rich sequence fragmented by guanine-to-adenine substitutions (in red) was used as a negative control. (**g**) CD spectra were measured in the presence of 100 mM KCl at 25 °C and 65 °C. The CD spectrum of the negative control was measured at 25 °C. Data represent the mean ± s.e.m. independent-sample *t*-test, *p < 0.05, **p < 0.01, ***p < 0.001.

It was reported that a two-SNP haplotype (rs10420331-rs11084307) in the intronic region of *CACNG8* was associated with schizophrenia^29^. Since these two SNPs do not alter the γ-8 protein sequence, we suspected that they might regulate the gene expression level. To test this possibility, we created a GFP reporter system by fusing a γ-8 genome sequence containing partial exons and an intron region including different SNPs to GFP cDNA controlled by the EF1α promoter (Fig. 1a). The reporter vectors were transfected into HEK293T cells, and GFP expression was analyzed by western blot. Neither rs10420331(G/A) nor rs1084307(T/C) affected the expression level of the GFP reporter protein (Fig. 1b,c), indicating they may not affect the expression of TARP γ-8.

Since closely localized SNPs, such as those within a single intron, often exhibit genetic linkage, we wondered whether the TARP γ-8 expression levels could be controlled by other *CACNG8* SNPs, rather than the previously reported ones. There are 6 high-frequency (> 0.05) SNPs in *CACNG8* (rs10420324, rs11084307, rs8111479, rs4806677, rs34012356, and rs10420331). rs10420324, rs11084307, rs8111479 and rs10420331 are in intron 1, while rs34012356 is in intron 2 and rs4806677 is in intron 3. Their variation in the Chinese Han Beijing (CHB) population was identified according to the SNPinfo web server^30^ (Fig. 1d). The results show that some SNPs have high linkage disequilibrium (LD) values, indicating that these SNPs are genetically linked directly or indirectly.

To determine the effects of the six SNPs on gene expression, reporters for each SNP were constructed using the same strategy shown in Fig. 1a. The expression of the GFP reporter protein was approximately 50% lower in the cells transfected with rs10420324G than in those transfected with rs10420324T, while the other 5 pairs of SNP variations had no effect on GFP expression (Fig. 1b,c). To further determine the effects on expression, the pre-mRNA and mRNA expression levels of the GFP fusion construct were determined by RT-PCR using specifically designed primers (Fig. 1a, bottom panel). The pre-mRNA and mRNA expression levels were lower in the cells transfected with rs10420324G than in those transfected with rs10420324T (Fig. 1e), indicating that the transcription of the reporter fusion constructs was regulated by rs10420324. Together, these results indicate that TARP γ-8 expression levels could be controlled by rs10420324, rather than the previously reported ones. It should be noted that rs10420324 is highly linked (LD = 0.7529) to the reported rs10420331^29^.

### SNP rs10420324 regulates G-quadruplex conformation and stability

The SNP rs10420324 is located in a guanine-rich region, and the local sequence likely forms a G-quadruplex secondary structure, a stack of planar four-guanine units called G-quartets, that affects gene expression by blocking the progression of RNA polymerase^31,32^. To analyze G-quadruplex formation and stability, two oligonucleotides containing rs10420324(G/T) and one control oligonucleotide with guanine-to-adenine substitutions were synthesized (Fig. 1f) and analyzed by circular dichroism (CD). The CD wavelength of rs10420324T at 25 °C showed a positive Cotton effect near 265 nm and a negative one at approximately 240 nm (Fig. 1g), suggestive of a parallel-form G-quadruplex. The CD spectra of rs10420324G showed positive ellipticity near 265 nm, a strong shoulder at approximately 295 nm, and a negative one close to 240 nm, suggestive of a hybrid-form G-quadruplex, while the control oligonucleotide displayed no typical G-quadruplex waveforms (Fig. 1g). We then measured the G-quadruplex stability by analyzing the thermostability and heated each to 65 °C. The G-quadruplex waveform of rs10420324G was thermostable, with little change in the spectra, while the positive and negative ellipticities in the CD spectra of rs10420324T were significantly reduced by heating (Fig. 1g), suggesting that the G-quadruplex formed by rs10420324T was less stable. Taken together, these results demonstrate that rs10420324 affects the conformation and stability of a local G-quadruplex in *CACNG8*.

### SNP rs10420324G suppresses TARP γ-8 expression in the human brain

**Figure 2:**
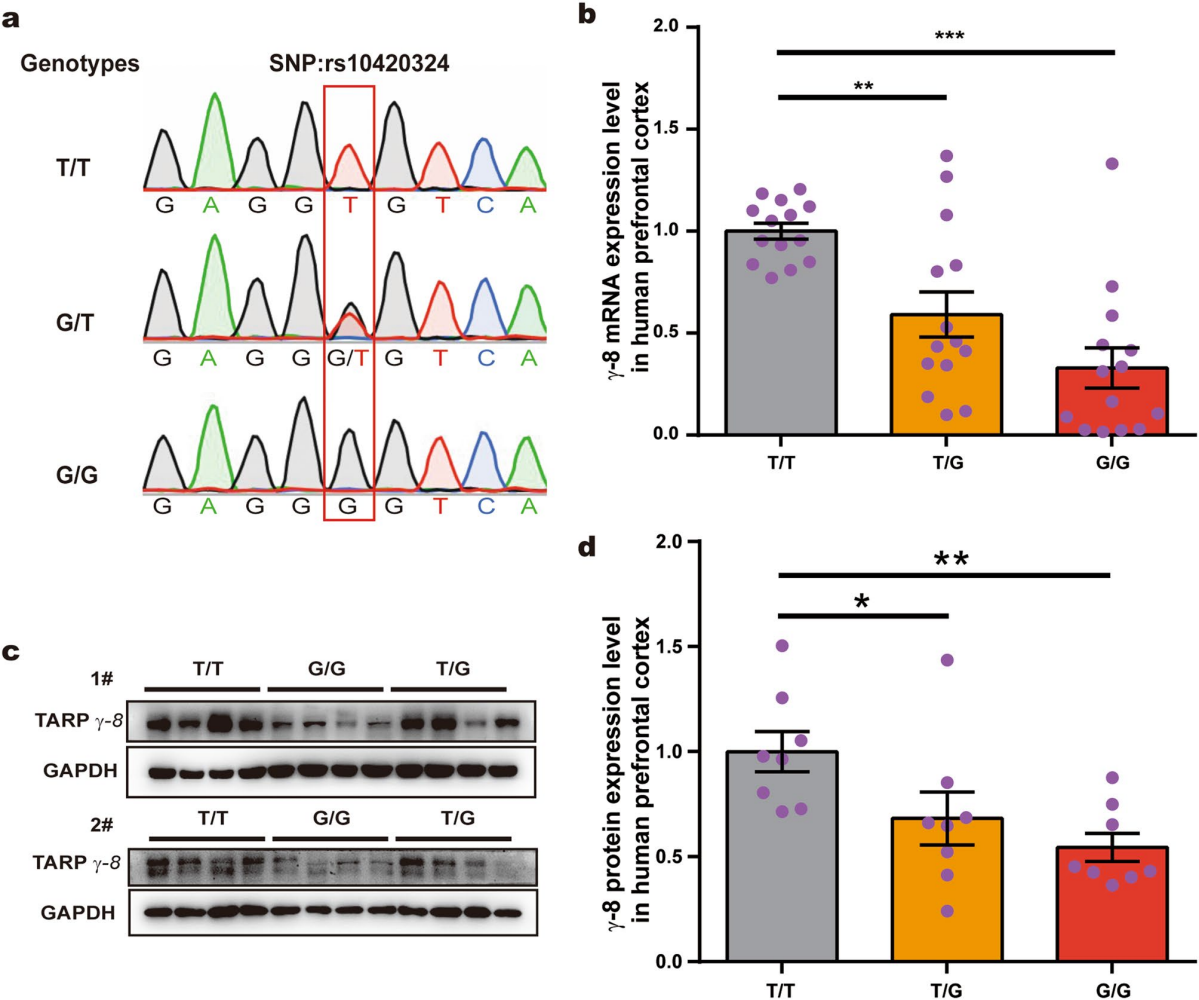
SNP rs10420324 determines TARP γ-8 expression in human brains. (**a**) Sanger sequencing results showing the T/T, G/T, and G/G genotypes at the CACNG8 rs10420324 locus in human prefrontal cortex tissues (outlined with a red box). 101 human prefrontal cortex tissues of male adults (25–45 years old) were obtained from a Chinese brain bank in Wuhan. (**b**) Fold change in the TARP γ-8 mRNA expression level in the human prefrontal cortex tissues with different rs10420324 genotypes (n = 14 for each). TARP γ-8 mRNA expression was normalized using β-actin mRNA. (**c**) Western blot analysis of the TARP γ-8 proteins in human prefrontal cortex tissues with different rs10420324 genotypes (n = 8 for each genotype). GAPDH was an internal reference. (**d**) Quantification of the TARP γ-8 protein expression levels in (**c**). TARP γ-8 expression was normalized using GAPDH. Data represent the mean ± s.e.m. one-way ANOVA, *p < 0.05, **p < 0.01, ***p < 0.001.

Next, we aimed to understand the effect of rs10420324 on TARP γ-8 expression. As the SNP rs10420324 has been found only in humans, we analyzed human prefrontal cortex tissues of 101 male adults (25–45 years old) from a Chinese brain bank in Wuhan. The samples were genotyped by Sanger sequencing, which identified 35, 52, and 14 samples with the T/T, G/T, and G/G genotypes at the rs10420324 site, respectively (Fig. 2a). Quantitative RT-PCR revealed that the mRNA expression level of TARP γ-8 was negatively correlated with the number of Gs at the rs10420324 site. Specifically, relative to that in T/T samples, the mRNA expression of TARP γ-8 was 59.2% in the G/T and 32.9% in the G/G samples (Fig. 2b, n = 14 for each genotype). Consistent with the mRNA expression levels, the protein levels of TARP γ-8 were also negatively correlated with the number of Gs at the rs10420324 site; relative to that in T/T samples, the protein levels of TARP γ-8 was 68.2% in the G/T and 54.4% in the G/G samples (Fig. 2c,d, n = 8 for each genotype). These results demonstrate that the SNP rs10420324G suppresses TARP γ-8 gene expression in the human brain.

### SNP rs10420324 is associated with ASPD

Above results indicate that the SNP rs10420324 regulates γ-8 expression levels in the human brain. We wondered whether this SNP is linked to psychological disorders in humans. We sequenced the rs10420324 site in 135 patients diagnosed with ASPD (25–40 years old) that were initially identified from male inmates from an adult male prison in Jiangsu Province^33^ and in 487 healthy controls (30–46 years old) by Sanger sequencing. There were significant differences in the genotypes (χ^2^ = 8.471, *p* = 0.014) and allele frequencies (χ^2^ = 8.537, *p* = 0.003) between the two groups; compared to the healthy control group, the ASPD group had an increased GG genotype frequency (30.4% vs. 19.7%) and G allele frequency (53.3% vs. 43.3%; Tables 1, 2). It should be noted that the rs10420324 G/T distribution in the control group was consistent with documented data in the CHB population. Thus these data indicate that the rs10420324G is associated with ASPD in Chinese Han samples.

**Table 1:** Genotype frequency difference between the ASPD and control groups.

Group	n	Genotype frequency (%)			χ^2^	*p*
rs10420324		GG	TG	TT		
ASPD	135	41 (30.4)↑	62 (45.9)	32 (23.7)↓	8.471*	0.014
Controls	487	96 (19.7)	230 (47.2)	161 (33.1)		

*ASPD* antisocial personality disorder.

*Intergroup comparisons were tested by chi-square test.

**Table 2:** Allele frequency difference between the ASPD and control groups.

Group	n	Allele frequency (%)		χ^2^	*p*
rs10420324		G	T		
ASPD	270	144 (53.3)↑	126 (46.7)↓	8.537**	0.003
Controls	974	422 (43.3)	552 (56.7)		

*ASPD* antisocial personality disorder.

**Intergroup comparisons were tested by chi-square test.

### TARP γ-8 knockout mice exhibit increased aggression and impulsivity

**Figure 3:**
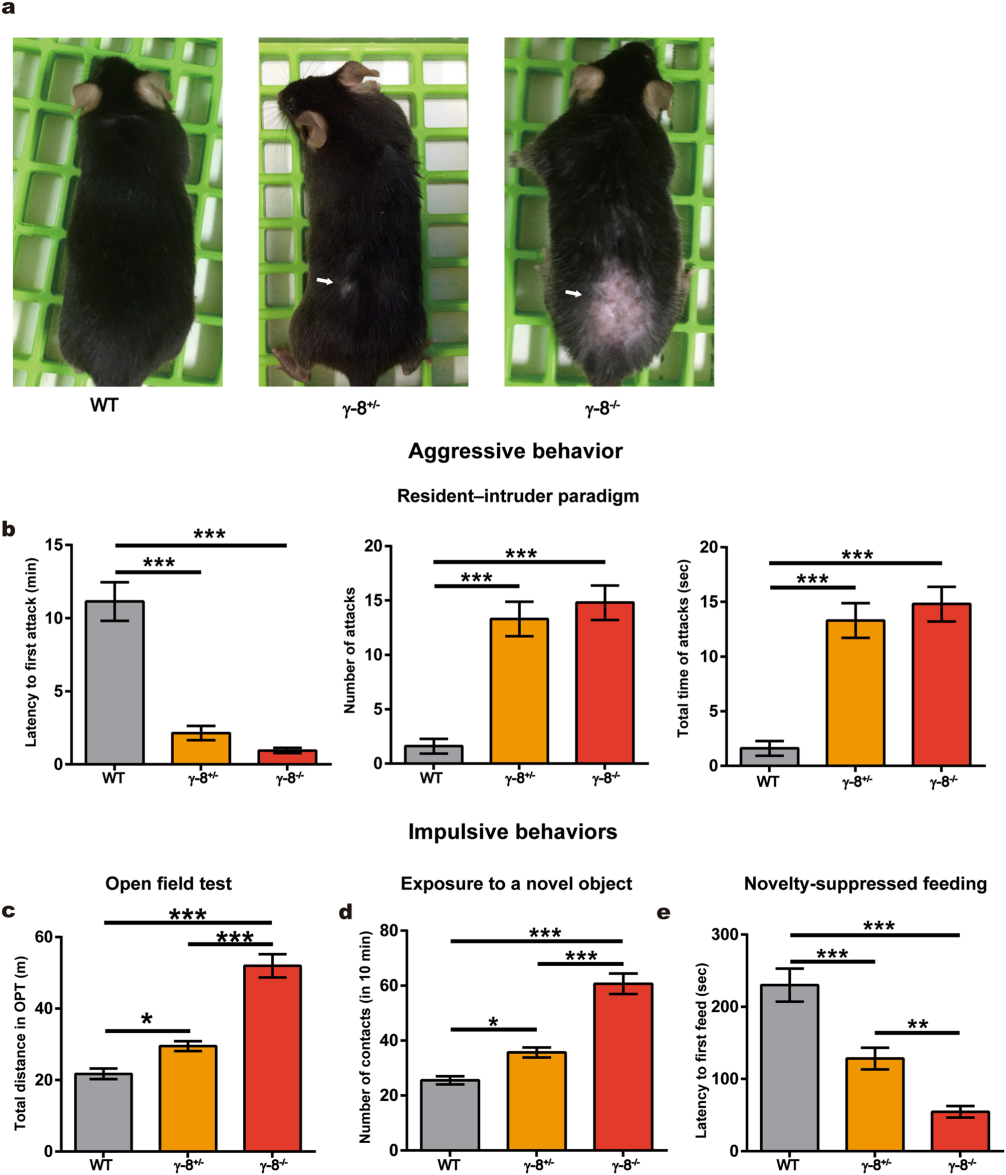
Aggression and impulsivity of WT and mutant mice. (**a**) Representative photos of WT and mutant mice at three-month-old. Four same genotype mice were housed in one cage. The arrows indicate areas where the animal was bitten. (**b**) Aggressive behavior test (resident-intruder paradigm). The latency to the first biting attack (left), the total number of attacks (middle) and the total duration of attack episodes (right) are presented. n = 10 cages (four mice in each cage). (**c**–**e**) Impulsive behavior tests. (**c**) Open field test. Total distance travelled in 5 min. n = 12. (**d**) The number of contacts with a novel object in 10 min. n = 12. (**e**) Novelty-suppressed feeding test. Degree of hyponeophagia in WT and mutant mice following an 18-h fast. n = 12. Data represent the mean ± s.e.m. one-way ANOVA, *p < 0.05, **p < 0.01, ***p < 0.001.

To understand the causal relationship between the decrease of TARP γ-8 and ASPD, we designed systematic neurobehavioral tests and analyzed the behavior of γ-8^−/−^ and γ-8^+/−^ mice^27^, in which either both or one of the γ-8 alleles were knocked out, respectively. The γ-8^−/−^ and γ-8^+/−^ mice exhibited bare patches in their coats from severe bite wounds, suggesting that these mice were more aggressive than wild-type (WT) animals, which did not have bite wounds (Fig. 3a). For confirmation, we used the resident–intruder paradigm test to evaluate aggression^34,35^, which is a standardized method to measure offensive aggression and defensive behavior in a semi natural setting^35^. The experiments were performed and analyzed by persons blinded to the mouse genotypes. As expected, the γ-8^−/−^ and γ-8^+/−^ mice exhibited a shorter latency to the first biting attack, a higher attack frequency and longer total duration of attack episodes than WT mice (n = 10, Fig. 3b), demonstrating that reduced γ-8 expression led to aggressive behaviors in mice.

Next, the general behavioral characteristics of the γ-8^−/−^ and γ-8^+/−^ mice were investigated using the open field test. We found that the total distance traveled was greater for γ-8^−/−^ and γ-8^+/−^ mice than for WT mice, and the total distance of γ-8^−/−^ mice higher than γ-8^+/−^ mice (n = 20, Fig. 3c), while the time in central vs. periphery was not different among γ-8^−/−^, γ-8^+/−^ and WT mice (n = 20, Fig. S1). Those results suggested that reduced γ-8 expression caused an increased locomotor response to environmental novelty, a phenotype classically associated with impulsivity^36^. We then further examined impulsivity by testing the response to novelty. A novel object (a blue plastic cap from a 15 mL Falcon tube) was introduced into the same corner of the home cage of each mouse. The number of physical and nonphysical (sniffing) interactions (referred to as contacts) of the animal with the object were recorded for 10 min. Compared with the WT mice, both γ-8^−/−^ and γ-8^+/−^ mice (n = 12) interacted more with the novel object (n = 12, Fig. 3d), indicating again that the mutant mice were more impulsive than the control mice. Impulsivity was further analyzed with a novelty-suppressed feeding test, which examines the consequences of competing motivations, i.e., the drive to eat versus fear of venturing into the center of a brightly lit arena where the food is located^36^, known as hyponeophagia. Mice were fasted for 18 h and then placed in a new cage with a brightly lit arena containing a pellet of food. The latency to start eating (defined as the mouse sitting on its haunches and biting the pellet while holding it with the forepaws) was recorded within a 5 min period. Compared to WT mice, γ-8^−/−^ and γ-8^+/−^ mice (n = 12) had shorter eating latencies and γ-8^−/−^ mice shorter than γ-8^+/−^ mice (Fig. 3e), also indicating reduced γ-8 expression led to impulsive behaviors in mice.

The resident–intruder paradigm test, the open field, novel object exposure and novelty-suppressed feeding tests suggested that γ-8 expression is negatively associated with aggression and impulsivity in mice.

### TARP γ-8-null mice show increased antisocial and risk-igonoring behaviors

**Figure 4:**
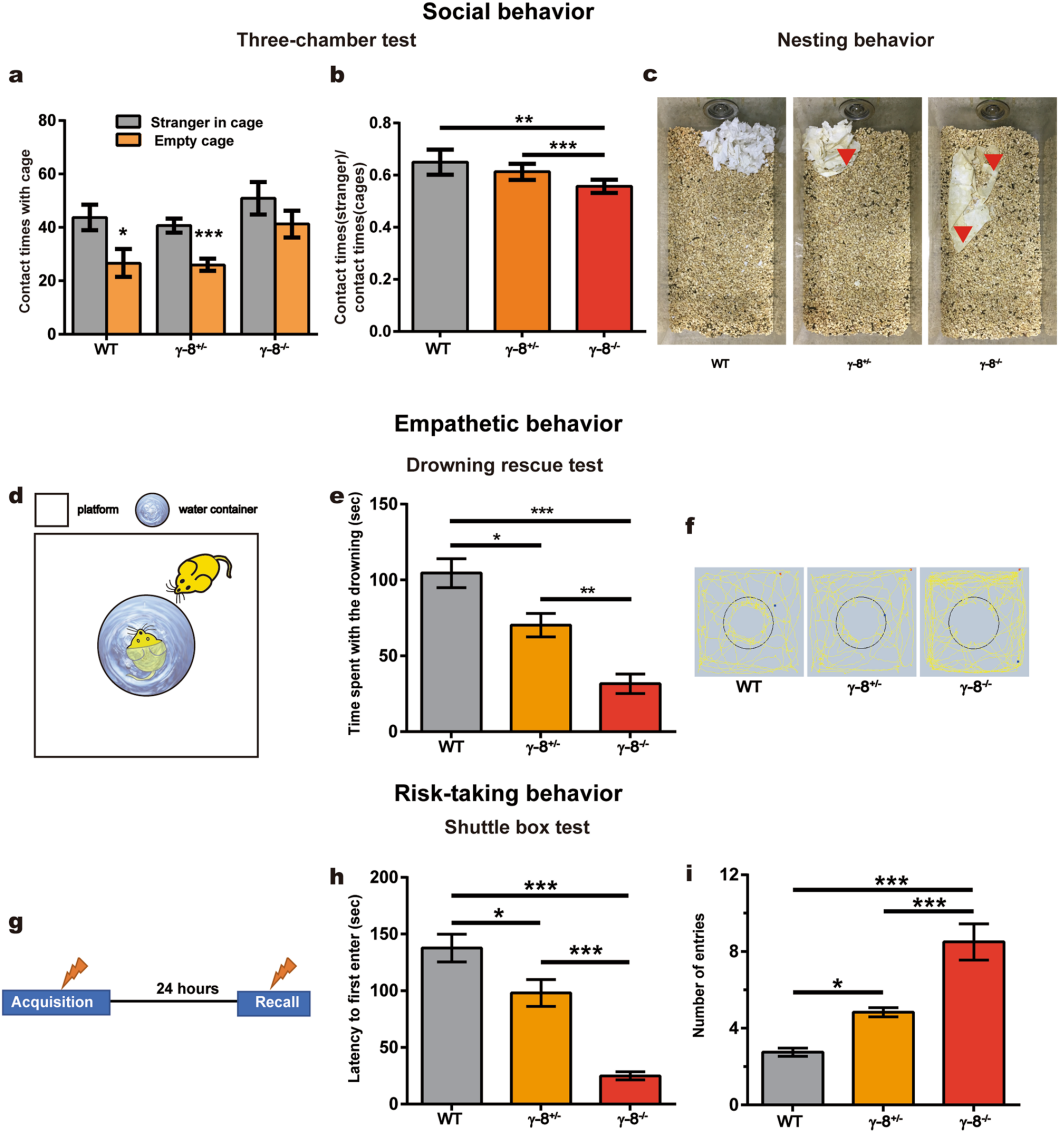
Impaired prosocial behavior and lesson-learning ability in TARP γ-8 knockout mice. (**a**, **b**) Three-chamber tests. (**a**) Contact times with the empty cage or stranger cage during the 10 min period and (**b**) stranger/total contacts of the WT (n = 11), TARP γ-8^+/−^ (n = 12) and TARP γ-8^−/−^ (n = 11) mice. Data represent the mean ± s.e.m. Independent-sample *t*-test used in (**a**), one-way ANOVA used in (**b**), *p < 0.05, **p < 0.01, ***p < 0.001. (**c**) Representative photos of mouse cages taken after four mice of same genotype housed in one cage for 7 days without a change of bedding. The paper was the nest where the animals slept. The arrows indicate yellow stains on the paper from the urine of TARP γ-8^+/−^ and TARP γ-8^−/−^ mice while no yellow stains on the nest were observed in the cages of the WT mice. (**d**–**f**) Empathetic behavior tests. (**d**) Schematic of the drowning rescue test. The mice in platform were the cage mate of mice in the water container. (**e**) Time spent with the drowning cage mate during the 5 min period and (**f**) movement traces of the WT, TARP γ-8^+/−^ and TARP γ-8^−/−^ mice (n = 12 for each) in the drowning rescue test. Red dots: experiment start position, Blue dots: experiment end position, Yellow line: movement traces, the black circle: 4 cm from the edge of the container. (**g**–**i**) Risk-taking behavior test. (**g**) Schematic of the shuttle box test. After receiving a plantar shock (defined as acquisition), mice were fed freely for 6 h and then fasted for 18 h. The mice were tested for 180 s. Lightning bolts: Bottom shock. (**h**) initial latency to enter and (**i**) total number of entries (n = 12 for each). Data represent the mean ± s.e.m. one-way ANOVA, *p < 0.05, **p < 0.01, ***p < 0.001.

To furtherly investigate the potential relationship between TARP γ-8 and mood disorders, we used a series of behavioral tests to measure different endophenotypes of emotional disorders: social behavior, empathy-related behavior and risk-taking behaviors^37–39^. We first tested their social behaviors by three-chamber test. The test mouse was free to explore the apparatus, and the preference to contact the stranger mouse placed inside a wire cage vs. an empty wire cage placed in the opposite chamber was assessed. Whereas the WT and γ-8^+/−^ mice preferred to stay with the stranger mice, γ-8^−/−^ mice showed no preference (Fig. 4a,b). These results indicate that γ-8^−/−^mice have social preference injury.

In addition, in contrast to the WT mice, the γ-8^−/−^ and γ-8^+/−^ mice showed little or no nesting behavior, defined as crushing the paper and piling the pieces together to make a fluffy nest (Fig. 4c). Furthermore, the γ-8^−/−^ and γ-8^+/−^ mice urinated throughout their cages, even on their nest (Fig. 4c, yellow spots marked with arrows); thus, their cages were much dirtier than those of WT mice. We hypothesized that these reductions in cage management behaviors in γ-8^−/−^ and γ-8^+/−^ mice indicated reduced social responsibility.

To further understand the social responsibility of these mouse genotypes, we designed an experiment called the drowning rescue test (Fig. 4d) to examine their helping behavior. In this experiment, a mouse was placed in a water-filled container measuring 15 cm in diameter in the center of a 60 × 60 cm^2^ field. A cage mate was then introduced into the field outside of the container, and the level of interaction with the container containing the struggling-against-drowning mouse were monitored. The WT mice spent more time near the container (defined as keeping their own center of gravity less than 4 cm from the edge of the container) than did the γ-8^−/−^ and γ-8^+/−^ mice, indicating empathetic behavior (Fig. 4e). Similarly, WT mice had significantly more contacting movement traces than gene deficient mice (Fig. 4f). These data suggest that compared to the WT mice, the γ-8^−/−^ and γ-8^+/−^ mice display callousness-like behaviors, which is classically associated with antisocial behavior^40,41^.

Antisocial behavior often includes elevated risk-taking behaviors^42^. To evaluate risk managing behavior, we used the shuttle box apparatus, which divided into two halves, one with an electric shock at bottom^43^. During the acquisition trial, immediately after the fasted mice entered the food-containing compartment, a five-second, 30-V electric shock was delivered to the mouse (Fig. 4g). In a recall trial 24 h later, compared to the WT mice, the γ-8^−/−^ and γ-8^+/−^ mice exhibited a significantly reduced latency to enter the compartment (Fig. 4h) and a significantly increased frequency of entries in 5 min (Fig. 4i), revealing that the γ-8^−/−^ and γ-8^+/−^ mice did not learn to fear the shock lesson and thus exhibited risk-ignoring behaviors.

Three-chamber test, bedding management, the drowning rescue test and shuttle box test suggested that γ-8 expression is negatively associated with social impairment, irresponsibility-like behaviors, callousness-like behaviors and risk-ignoring behaviors.

### TARP γ-8 knockout impairs synaptic AMPAR function in medial prefrontal cortex

**Figure 5:**
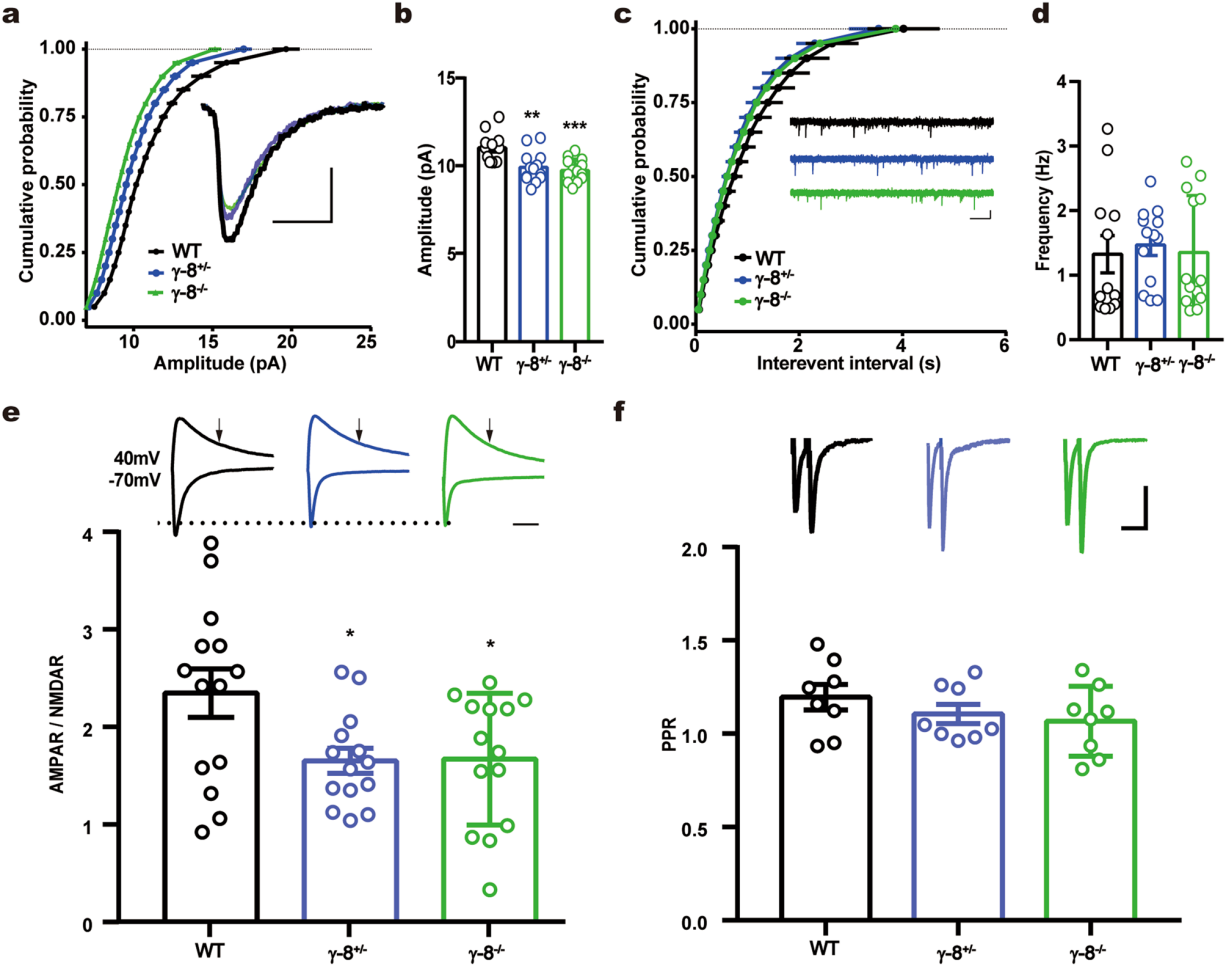
Synaptic AMPAR functions are impaired in mPFC TARP γ-8 knockout mice. (**a**–**d**) mEPSCs from γ-8^+/−^ and γ-8^−/−^ mPFC pyramidal cells had reduced amplitude but unchanged frequency. (**a**) Cumulative frequency distribution of mEPSC amplitudes. Insert shows sample traces. Scale bar, 5 pA, 5 ms. (**b**) Amplitude of mEPSC was reduced in mPFC pyramidal cells from γ-8^+/−^ and γ-8^−/−^ mice. (**c**) Cumulative frequency distribution of mEPSC interevent interval. Sample traces are shown as insert. Scale bar, 10 pA, 500 ms. (**d**) Frequency of mEPSC did not differ among WT, γ-8^+/−^ and γ-8^−/−^ neurons. (n = 12 for WT; n = 13 for γ-8^+/−^; n = 13 for γ-8^−/−^). (**e**) AMPA/NMDA ratio was reduced in mPFC pyramidal neurons from γ-8^+/−^ and γ-8^−/−^ mice (n = 14). Representative EPSCs recorded at holding of − 70 mV and + 40 mV are shown above the bar graph. Arrows indicate where NMDAR-EPSCs were measured. Scale bar, 50 ms. (**f**) Paired-pulse ratio (PPR) does not differ between WT, TARP γ-8^+/−^ and TARP γ-8^−/−^ neurons (n = 8 for each). The sample traces are shown above the bar graph. Scale bar, 50 pA, 100 ms. Data represent the mean ± s.e.m. one-way ANOVA, *p < 0.05, **p < 0.01, ***p < 0.001.

The medial prefrontal cortex (mPFC) is a brain region that is liked to aggressive behaviors in humans and in rodents^44^; lesion or inhibition leads to aggression. Since TARP γ-8 was known to regulate AMPAR functions, we thus chose to analyze synaptic AMPAR function in the layer II/III pyramidal neurons of mPFC in γ-8^−/−^ and γ-8^+/−^ mice. The amplitude of miniature excitatory postsynaptic currents (mEPSCs) was significantly reduced in γ-8^−/−^ and γ-8^+/−^ neurons compared to WT neurons (Fig. 5a,b) while mEPSC frequency was not different among genotypes (Fig. 5c,d). When EPSCs were evoked by stimulating layer IV with a bipolar tungsten electrode, the AMPA to NMDA ratio was reduced in γ-8^−/−^ and γ-8^+/−^ mices compared to WT (Fig. 5e). Paired pulse ratio (PPR) of AMPAR EPSCs evoked by a pair of stimuli with 50 ms intervals was not different among WT, γ-8^−/−^ and γ-8^+/−^ (Fig. 5f), indicating neurotransmitter release probability was unaltered by γ-8 expression level.

## Discussion

**Figure 6:**
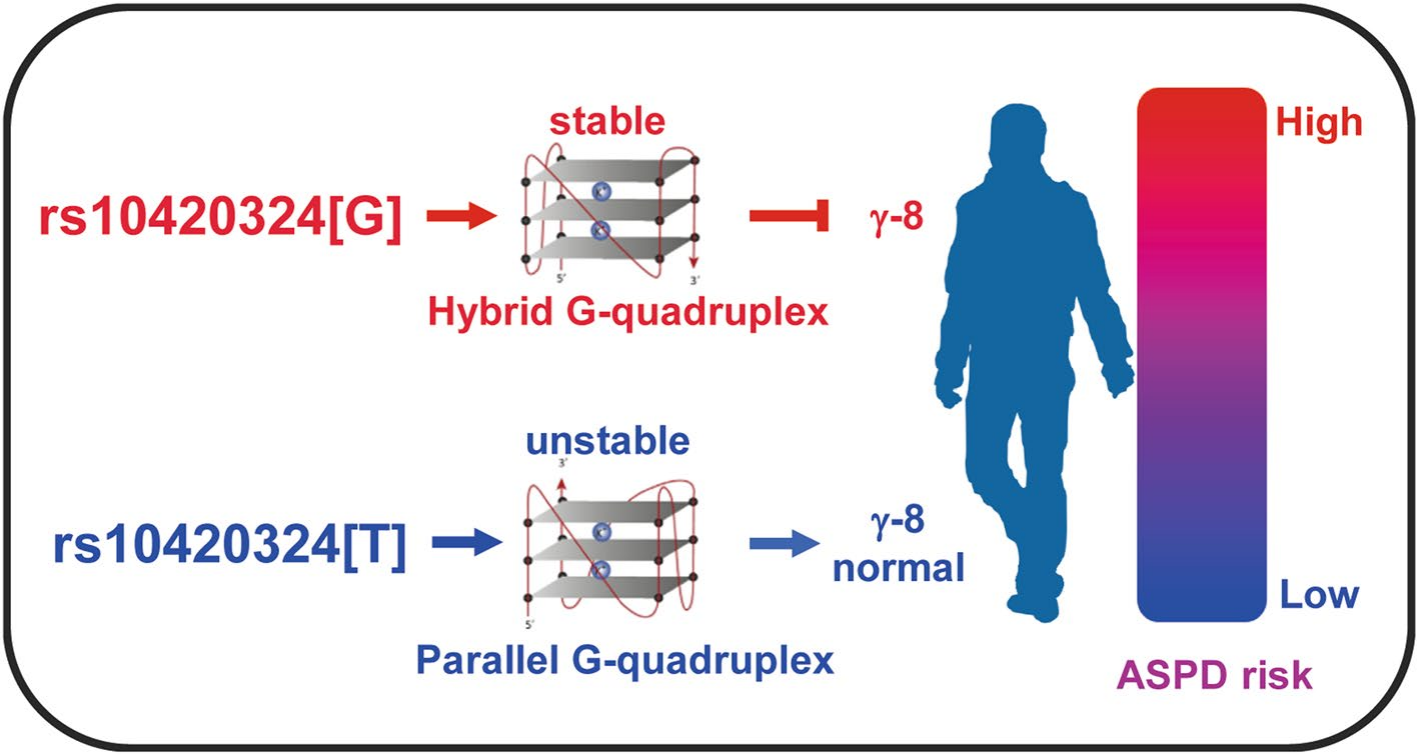
A model illustrating a molecular mechanism that rs10420324 in *CACNG8* regulates the susceptibility to ASPD. The SNP site rs10420324 (T/G) in the 1st intron of *CACNG8* determined TARP γ-8 expression by regulating the conformation (parallel/hybrid) and stability of a local G-quadruplex. The rs10420324G suppresses TARP γ-8 expression, thus increases ASPD risk.

In this study, we aim to understand the role of the AMPA receptor regulatory protein TARP γ-8 in emotional disorders. Our study shows that the expression level of γ-8 contributes to ASPD in humans. This conclusion is supported by the evidence from ASPD patients and mouse models. In human, the SNP site rs10420324 in the 1st intron of *CACNG8* determines the transcription of TARP γ-8, likely by regulating the conformation and stability of a local G-quadruplex. Compared to rs10420324T, rs10420324G suppresses transcription. The rs10420324G is higher in patients diagnosed with ASPD than in controls, indicating that lower TARP γ-8 expression is associated with ASPD. In mice models, compared to the WT, the TARP γ-8 mutant mice exhibited greater impulsivity, aggressiveness, risk-ignoring, irresponsibility-like and callousness-like behaviors, all of which are psychological characteristics linked to ASPD in humans^45^. Reduction in TARP γ-8 impairs synaptic AMPAR function in mPFC, a brain region linked to aggression^44^. Our study suggest a model shown in Fig. 6.

A previous study identified a two-SNP haplotype (rs10420331-rs11084307) in associated with schizophrenia^29^. These two SNPs, however, do not affect the expression of the reporter GFP, indicating they cannot regulate γ-8 expression. In contrast, SNP rs10420324, at a nearby locus, significantly affected the transcription of the GFP reporter molecule. SNP rs10420324 is located in guanine-rich nucleic-acid sequences, which can form non-canonical structure G-quadruplexes^46^. Substantial evidence now exists to support that formation of DNA or RNA G-quadruplexes is coupled to altered gene expression by blocking the progression of RNA polymerase^46–48^. The variants of rs10420324 regulates the conformation and stability of the local G-quadruplex structure. The stable hybrid G-quadruplex formed by rs10420324G can suppress reporter gene transcription in vitro and TARP γ-8 transcription in vivo, supporting the notion that the G-quadruplex regulates transcription. Our observations provide strong evidence in G-quadruplexes formation linking with biological processes of transcription (Fig. 6).

TARP γ-8 is highly expressed in the hippocampus, cortex and subcortical regions that are critical for emotion generation in humans^19^. Genetic deletion of TARP γ-8 led to reduced AMPARs on the neuronal surface^26^ and impaired synaptic AMPA-EPSCs and Long-term potentiation (LTP)^27^. LTP is a persistent strengthening of synapses and widely considered one of the major cellular mechanisms that underlies learning and memory, recognition and emotion^49,50^. Recent studies demonstrated that LTP induction required the PDZ binding motif and CaMKII phosphorylation sites located at the C-terminal region of γ-8^51,52^. It has been reported that γ-8 knockout mice showed hyperactivity^28^, a phenotype we also observed in this study. Interestingly, chemical interference with TARP γ-8 function also led to hyperactivity in rats^53^, indicating that TARP γ-8 is a valid drug target. Most importantly, we observed that the γ-8^−/−^ and γ-8^+/−^ mice had multiple behavioral abnormalities that likely mimicked antisocial behaviors associated with ASPD in humans.

ASPD is one of the leading risk factors associated with criminal behaviors, such as violent crime and murder, in humans^54^. Impulsiveness, lack of foresight, and aggression are the most direct causes of crime, inflicting harm on both the agent and society^55–57^. Individuals with ASPD often engage in dangerous, risky, and potentially self-damaging activities unnecessarily and without regard for consequences^58^. In our study, γ-8-null mice displayed many behaviors mimicking the above mentioned features of human ASPD. Firstly, γ-8^−/−^ and γ-8^+/−^ mice displayed elevated aggression with enhanced attacking behavior against intruders and engaged in intense fighting and biting between cage mates. Secondly, these mice had greater impulsivity: they did not examine their surroundings for safety when faced with novel objects or were presented with food in novel environments. In addition, these animals showed a reduced lesson-learning ability and risk-ignoring behavior when facing danger, similar to ASPD patients, who often disdain laws and regulations, have little regard for punishment and benefit little from past punishment or other experiences^59,60^. Finally, individuals with ASPD tend to show diminished responsibility and empathy^45,58^. ASPD can be identified according to the presence or absence of callous-unemotional traits (deficits in empathy and guilt)^61^. TARP γ-8^−/−^ and γ-8^+/−^ mice failed to build nests or manage their cages, indicating irresponsibility-like behaviors. These mice were also indifferent to their drowning cage mates, indicating that a decrease in TARP γ-8 expression impairs empathetic behavior in mice. Interestingly, the severity of all the ASPD-associated behaviors observed in this study was dependent on the γ-8 expression level, with the γ-8^−/−^ mice having a more severe phenotype than the γ-8^+/−^ mice. Notably, to our knowledge, there is no available animal model of ASPD. Therefore, γ-8 mutant mouse is a valuable animal model for studying ASPD.

Using mutant mice and human samples, we have demonstrated that the expression level of the AMPAR regulatory subunit TARP γ-8 contributes to multiple behaviors linked to ASPD (Fig. 6). Our study lays a key foundation for future study of ASPD at least in following two aspects. Firstly, the TARP γ-8 knockout mice is an appropriate animal model for ASPD study. This animal model will setup a start point to dissect the neural circuits related to ASPD. Then, TARP γ-8 and AMPARs are potential therapeutic targets for ASPD.

## Materials and methods

All experiments, statistical analyzes, and information provided in this article were made in accordance with the recommendations of the ARRIVE guideline.

### Selection of SNP markers

Four tag SNPs (rs10420324, rs11084307, rs8111479, and rs34012356) and two of their linked SNPs (rs4806677 and rs10420331) in *CACNG8* were selected. Their variation and the linkage disequilibrium (LD) in CHB populations was determined according to the SNPinfo web server^30^. population: CHB; Minor allele frequency: > 0.05; LD threshold: > 0.8; Sort by LD similarity.

### Cell culture and transfections

HEK 293 T cells were cultured in Dulbecco’s Modified Eagle Medium (DMEM) supplemented with 10% FBS and penicillin/streptomycin (Thermo Fisher Scientific) in a humidified incubator at 37 °C with 5% CO_2_. The culture medium was replenished every other day. HEK293T Cell were seeded in 24-well plates or 12-well plates. The HEK 293 T cells were transfected using Lipofectamine 2000 (11668030, Invitrogen) according to manufacturer’s instruction. Cells were collected for analysis 48 h after transfection.

### Participants and ethical approval

We recruited 2300 Chinese Han men: 1804 inmates from a prison^33^ and 496 healthy controls from the communities in Jiangsu Province, China. All procedures were approved by the Institutional Review Board of Nanjing Brain Hospital and a consent form for this study was prepared. We carried out the study in accordance with the principles of the Declaration of Helsinki. Individuals who had previously been diagnosed with chronic heart, liver and kidney diseases; had a history of nervous system diseases or mental disorders; or had a long history of medication use were excluded from the study. After informed consent was obtained, psychiatrists clinically assessed and diagnosed participants with ASPD using the self-reported Personality Diagnostic Questionnaire-4 (PDQ-4) and the Structured Clinical Interview Tool for DSM-IV Axis II Disorders (SCID-II). Among all participants, based on the ASPD scores in the PDQ-4 (ASPD score ≥ 4) and SCID-II (SCID-IIA score ≥ 3 or SCID-IIC score ≥ 2), 135 ASPD subjects and 487 healthy control subjects were ultimately included in this study.

### Human tissues

101 human prefrontal cortex (PFC) brain tissues were provided by a Chinese brain bank in Wuhan collecting the tissues of body donors. The tissues used in this study were all from male adults (25–45 years old).

### Genotyping

DNA was extracted from 5 ml of venous blood per person from 135 men with ASPD and 487 healthy men by the modified SDS method. PCR amplification (Taq DNA Polymerase, Vazyme) and Sanger sequencing (Genscript, China) were used for genotyping of rs10420324. The primers for genotyping were as follows: F: 5′-GCCTCTCCTGTGGAAGTTTGAG-3′; R: 5′-CCTCTTACTCCTACGTTCTCCG-3′. rs10420324 was successfully genotyped in all 135 ASPD subjects and all 487 healthy men.

### Western blots

Proteins were extracted from HEK 293 T cells or human prefrontal cortex brain tissues using tissue total protein lysis buffer (Beyotime, Shanghai, China) for each assay. Proteins were electrophoretically separated on SDS-PAGE gels and subsequently immunoblotted onto nitrocellulose membranes that were blocked in skim milk and incubated at 4 °C for 16 h using antibodies against GFP (1:5000, ab290, Abcam), β-tubulin (1:10,000, BS1482M, Bioworld), TARP γ-8 (1:500, abs132666a, Absin) and GAPDH (1:10,000, 60,004–1-Ig, Proteintech). Detection was carried out using the horseradish peroxidase-conjugated secondary antibodies goat anti-mouse IgG (1:10,000, BS12478, Bioworld) and goat anti-rabbit IgG (1:15,000, BS13278, Bioworld) along with a High-sig ECL kit (Tanon, Shanghai, China), and signals were recorded using a Tanon 5200 multi automatic chemiluminescence image analysis system (Tanon, Shanghai, China). For western blot quantitation, images were analyzed using ImageJ and expressed as arbitrary units (AU). GFP protein expression was normalized to β-tubulin protein expression, and TARP γ-8 expression was normalized to GAPDH expression.

### Quantitative real-time PCR analysis

Quantitative real-time PCR was performed to examine the relative expression levels of GFP in HEK 293 T cells and CACNG8 in the PFC of the human brain using an ABI Prism 7300 sequence detector (ABI, Massachusetts, USA). The following primer sequences were used: primer 1(for qPCR detecting fusion GFP pre-mRNA) of fusion GFP forward(F1): 5′-TGCTGCCTGGAAGGGTTGA-3′, reverse (R1): 5′-TGCCGTTCTTCTGCTTGTCG-3′; primer 2 (for qPCR detecting fusion GFP mRNA) of fusion GFP forward (F2): 5′-CAGTGCTTCAGCCGCTACCC-3′, reverse (R2): 5′-AGTTCACCTTGATGCCGTTCTT-3′; CACNG8 forward: 5′-GCGAGGGCTGAAGGGTAGTGA-3′, reverse: 5′-GCAATGGGTGAGTGGCTGAAT-3′; β-actin forward: 5′-AGCGAGCATCCCCCAAAGTT-3′, reverse: 5′-GGGCACGAAGGCTCATCATT-3′.Total RNA was extracted from HEK 293 T cells or the PFCs of human brains using TRIzol Reagent (Invitrogen, California, USA) for each assay. RNA was converted to cDNA using a reverse transcription kit HiScript Q RT SuperMix for qPCR (Vazyme, Biotech). qPCR reactions were performed with AceQ qPCR SYBR Green Master Mix (Vazyme, Biotech) according to manufacturer’s instruction. The relative expression levels of GFP or CACNG8 were normalized to the expression of β-actin.

### CD spectroscopy

DNA oligonucleotides (Genscript, Jiangsu, China) were dissolved in 1X TE buffer (10 mM Tris-HC, 1 mM EDTA, pH 7.4) with 100 mM KCl at a concentration of 5 μM. Before analysis, the oligonucleotides were heated to 95 °C for 5 min, slowly cooled to room temperature, and incubated overnight. The CD experiment was performed on a J-810 spectrophotometer (JASCO, Tokyo, JAPAN) using a quartz cell with a 1 mm optical path length and an instrument scanning speed of 50 nm/min with a response time of 1 s and over a wavelength range of 220–320 nm. Finally, three scans taken at 25 °C or 65 °C were averaged to obtain a representative CD spectrum for each condition.

### Mice

The TARP γ-8^−/−^ mice^27^ were a gift from Roger A. Nicoll of UCSF and were backcrossed with C57BL/6 J mice for at least 8 generations before these experiments. Four to six mice were housed in groups with a 12-h dark/light cycle and free access to food and water in accordance with the Regulations on Mouse Welfare and Ethics of Nanjing University, China. All procedures were conducted with the approval of the Model Animal Research Center of Nanjing University, Nanjing, China. The same genotype mice were housed in one cage. Two- to three-month-old male mice were used in all experiments. All behavioral tests and data analyses were carried out by different investigators, and the one who analyzed the data was blind to the genotypes of the mice.

### Behavioral tests

ZhengHua software (ZhengHua, Anhui, China) were used for recording the central locus of mice and analysis of all behavioral data. A special room was used to conduct all behavioral experiments separately. Mice were transferred to the room 2 h before the experiment. All experiments were performed in triplicate.

### Resident–intruder paradigm

An adult male WT mouse was used as an intruder. An intruder mouse was transferred into the residents’ cage (four males per cage), and attack episodes by the resident mice were recorded for 15 min. The intruder was typically attacked and defeated by the residents and showed submissive freezing behavior. Aggressive behaviors by the resident mice were scored; these behaviors were defined as biting, laterally attacking and displaying piloerection. The latency to the first attack bite, the frequency of bites and the duration of attack episodes were manually quantified. Data from four residents in the same cage were collectively analyzed as one unit.

### Open field test

The test arena consisted of a plastic-bottom plate (46.5 cm × 46.5 cm) and four surrounding plastic walls (35.0 cm high). A mouse was initially placed in a corner of the arena facing the center and then allowed to freely explore the open field for 5 min. The arena was cleaned with 70% EtOH between trials. The distance traveled (cm) by each mouse was quantified.

### Novelty-suppressed feeding

The testing apparatus consisted of a plastic box, 37 cm × 57 cm × 10 cm, directly illuminated by white light. The floor was covered with 2 cm of corncob bedding. Six same genotype mice were housed in one cage for 7 days. Eighteen hours before the test, food was removed from the cages. At the time of testing, a single pellet of standard mouse chow was placed at the center of the box. The animal was placed in a corner of the box, and a stopwatch was immediately started. The latency to start eating (defined as the mouse sitting on its haunches and biting the pellet while holding it with the forepaws) was recorded within a 5 min period.

### Exposure to a novel object

A novel object (a blue plastic cap from a 15 mL Falcon tube) was introduced in the same corner of the home cage of each mouse. The object was placed in the cage after the animal was momentarily removed into other cage. The mouse was then returned to the cage by gently setting it down in the corner opposite the novel object. Data recording started immediately, and the physical and nonphysical (sniffing) interactions of the animal with the object were recorded for 10 min. The latency to approach (measured as first sniffing) and the number of contacts were analyzed by a researcher blinded to the genotypes.

### Three-chamber test

The social test apparatus was an opaque acrylic box with two pull-out doors and three chambers. Each chamber was identical in size (40 × 20 cm), with the dimensions of the entire box being 63 cm × 42 cm × 21 cm. There was a 10-cm gap between adjacent chambers that could be opened or closed with the removable doors. The transparent wire cage (12 cm in height and 9.5 cm wide) equipped with the novel, stranger mouse was placed 2 cm away from the edge of the testing chamber to allow an interaction between the mice. The whole experiment was performed under low illumination and quiet conditions. The unfamiliar, stranger mouse was introduced into the wire cage in one side compartment, and an empty cage was placed in the opposite side compartment. The test mouse was introduced into the middle chamber and habituated for at least 5 min. The partitions were then removed, and the test mouse was permitted to explore all 3 compartments for 10 min. The entire process was recorded by a camera hanging 1 m above the apparatus. The relative positions of the empty cage and social cage were counterbalanced across test animals. The contact times with stranger or empty cage was recorded using the automated ZhengHua software.

### Risk-taking behavior

The apparatus contained a chamber with two compartments (dark/light), which were connected by a guillotine door^43^. The dark compartment contained a stainless steel grid floor that could deliver electric shocks. The mice were first fasted for 18 h before the acquisition trial. Each mouse was habituated to the light compartment for 180 s before the guillotine door was raised. Immediately after the mouse entered the dark compartment containing food, the guillotine door was closed, and a five-second electric shock of 30 V was delivered. The mouse was then returned to its home cage, fed freely for 6 h and then fasted for 18 h. The next day, after the mice were habituated for 180 s in the light compartment, the guillotine door was raised, and the mice were free to enter the electrically charged and food-containing dark compartment. The latency before the first entry and the number of entries were recorded in a 5 min observation period.

### Drowning rescue test

A mouse was placed into a 15 cm dimeter and 15 cm depth container filled with 25 °C water with a depth of 13 cm in the center of a 60 × 60 cm field. A cage mate was then introduced in the field, and its attempts to contact the drowning mouse were monitored for 5 min. Mice were considered to display empathetic behavior if they remained near the drowning cage mate (defined as keeping their central locus less than 4 cm from the edge of the container).

### Slice preparation and patch-clamp recordings

Coronal brain slices (300 µm) were prepared from 4- to 6-week-old C57BL6 male mice in ice-cold cutting solution containing (in mM): 210 Sucrose, 25 NaHCO_3_, 2.5 KCl, 7 MgSO_4_, 1.25 NaH_2_PO_4_, 1.3 Na-Ascorbic Acid, 10 glucose, and 0.5 CaCl_2_. After cutting, slices were allowed to recover for 30 min at 32 °C and stored at room temperature in artificial cerebrospinal fluid (ACSF) containing (in mM): 119 NaCl, 26.2 NaHCO_3_, 2.5 KCl, 1 NaH_2_PO_4_, 11 glucose, 1.3 MgSO_4_, and 2.5 CaCl_2_. All solutions were constantly oxygenated with 95% O_2_/5% CO_2_. Slices containing the mPFC were transferred to a submersion recording chamber, superfused with oxygenated ACSF at a speed of 1–2 ml/min. Whole-cell patchclamp recordings were performed using pipettes pulled from borosilicate glass capillaries (BF150-110-10, Sutter Instrument, USA) with resistances of 4–6 MΩ. We used Cs-methanesulphonate based internal solution containing (mM): 135 Cs-methanesulphonate, 8 NaCl, 10 HEPES, 0.3 EGTA, 5 QX-314, 4 MgATP, 0.3 Na_3_GTP and 0.1 Spermine-C14 (290–295 mOsm, pH 7.3–7.4). mEPSCs were recorded at − 70 mV in the presence of tetrodotoxin (0.5 M) and bicuculline (100 M). For evoked EPSCs, Layer IV inputs were stimulated by bipolar tungsten electrodes (Science Products). The stimulus was adjusted to evoke a measurable, monosynaptic eEPSC in both cells. We identified the mPFC layer II/III PNs by somatic location and size. AMPAR eEPSCs were measured at a holding potential of − 70 mV, and NMDAR eEPSCs were measured at + 40 mV 150 ms after the stimulus, at which point the AMPAR eEPSC has completely decayed. Paired-pulse ratios were measured by giving two pulses at a 50 ms interval and taking the ratio of the peak of the second eEPSC over the first eEPSC from an average of 15–30 sweeps. Data were acquired with a Multiclamp 200B amplifier, Digidata1550, and Clampe10 software. Signals were filtered at 2 kHz and digitized at 5 kHz.

### Statistical analysis

Statistical analysis was performed using Statistical Product and Service Solutions (SPSS) 22.0. The differences in demographic characteristics between the ASPD group and the control group were examined by performing Hardy–Weinberg equilibrium and chi-square tests. Group comparisons of genotypes and alleles were also achieved using chi-square tests. The independent-sample *t*-test was used for comparing the two treatment groups, and one-way ANOVA was used for comparisons among more than two groups. Data are presented as the means ± SEMs. *P* < 0.05 was set as the threshold for significance (*P < 0.05, **P < 0.01, ***P < 0.001).

## Supplementary Information

Supplementary Information.
