# Supplementary material for: SNP rs10420324 in the AMPA receptor auxiliary subunit TARP γ-8 regulates the susceptibility to antisocial personality disorder
Source: Sci Rep. 2021 Jun 7;11:11997. doi: 10.1038/s41598-021-91415-9 (PMC8184779; doi:10.1038/s41598-021-91415-9)
Supplement: Supplementary file 1 — Supplementary Information. [file 41598_2021_91415_MOESM1_ESM.pdf]

SNP rs10420324 in the AMPA receptor auxiliary subunit TARP γ-8 regulates the susceptibility to antisocial personality disorder

Shi-Xiao Peng, Yue-Ying Wang, Min Zhang, Yan-Yu Zang, Dan Wu, Jingwen Pei, Yansong Li, Jiapei Dai, Xiaoyun Guo, Xingguang Luo, Ning Zhang, Jian-Jun Yang, Chen Zhang, Xiang Gao, Na Liu[&], Yun Stone Shi[&]

Supplementary Information

Supplementary Figures 1-2

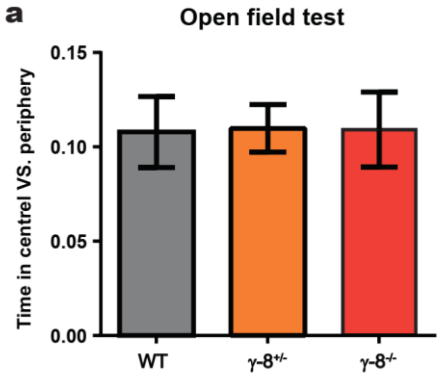

Supplementary Figure 1. Open field test. Time spend in central vs. time spend in periphery in 5 min. n = 12. There is no difference among γ-8$^{+/-}$, γ-8$^{-/-}$ and WT mice. The test arena consisted of a plastic-bottom plate (46.5 cm × 46.5 cm) and four surrounding plastic walls (35.0 cm high). The central 20 cm × 20 cm was defined as a central area. A mouse was initially placed in a corner of the arena facing the center and then allowed to freely explore the open field for 5 min.

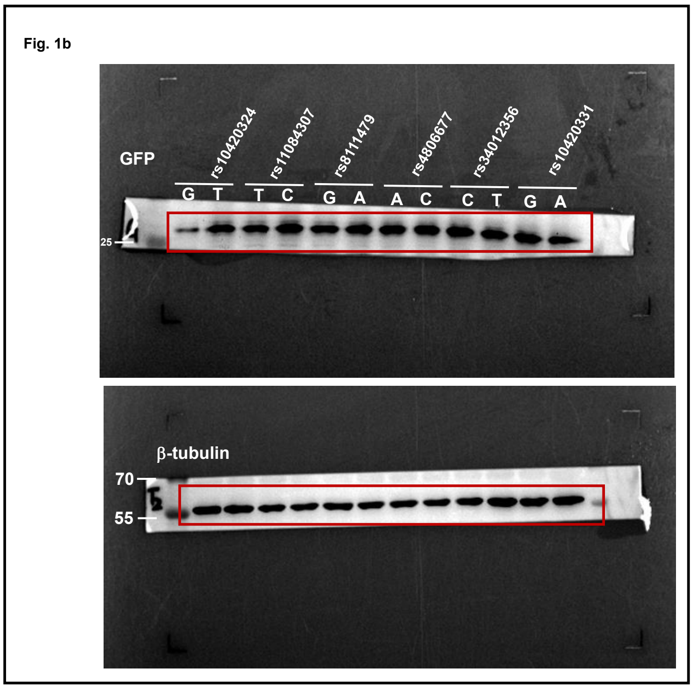

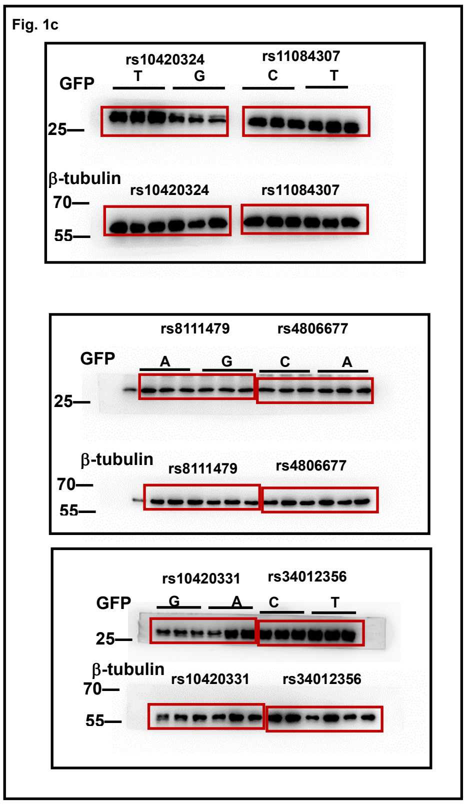

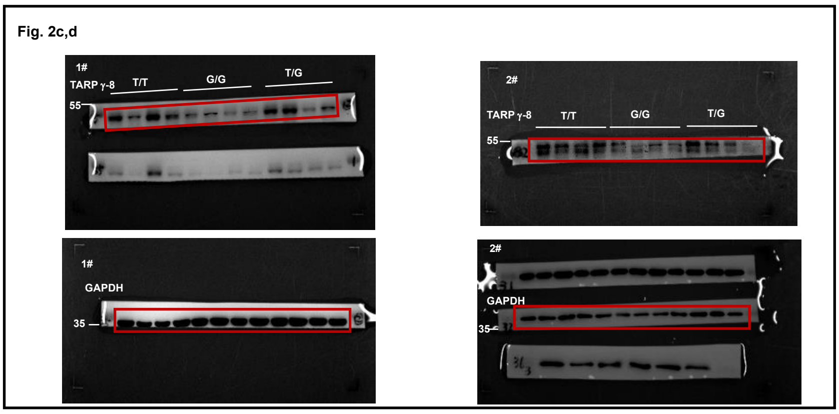

Supplementary Figure 2. The uncropped scans of western blots including the molecular weight markers for Figure 1b, Figure 1c, Figure 2c and Figure 2d as indicated.
